# Challenges of Surveillance in Implementing Nonoperative Management for Rectal Cancer

**DOI:** 10.1001/jamanetworkopen.2024.48682

**Published:** 2024-12-03

**Authors:** Bailey K. Hilty Chu, Anthony Loria, Totadri Dhimal, Xueya Cai, Shan Gao, Yue Li, Larissa K. Temple, Fernando Colugnati, Paula Cupertino, Erika E. Ramsdale, Fergal J. Fleming

**Affiliations:** 1 Surgical Health Outcomes and Reaching for Equity (SHORE), Department of Surgery, University of Rochester Medical Center, Rochester, New York; 2 Department of Biostatistics and Computational Biology, University of Rochester, Rochester, New York; 3 Department of Public Health Sciences, University of Rochester, Rochester, New York; 4 School of Medicine, Universidade Federal de Juiz de Fora, Juiz de Fora, Minas Gerais, Brazil; 5 James P. Wilmot Cancer Center, University of Rochester Medical Center, Rochester, New York

## Abstract

**Importance:**

Close surveillance for detection of local tumor regrowth is critical for patients opting for nonoperative management after neoadjuvant therapy for rectal cancer. However, there are minimal data regarding the feasibility and adherence to National Comprehensive Cancer Network (NCCN) surveillance guidelines for these patients.

**Objective:**

To determine adherence rates to NCCN-recommended surveillance in patients undergoing nonoperative management for rectal cancer.

**Design, Setting, and Participants:**

This retrospective cohort study included patients treated at a single academic center (University of Rochester, Rochester, New York) from 2012 to 2023, with analysis completed from March through May 2024. Patients with clinical stage I to III rectal cancer who underwent treatment with neoadjuvant therapy, achieved a clinical complete response (or initially near complete clinical response), and elected for nonoperative management were enrolled.

**Exposure:**

Achievement of recommended surveillance.

**Main Outcomes and Measures:**

The primary outcome was adherence to surveillance guidelines for patients undergoing nonoperative management for rectal cancer. Secondary outcomes included assessment of oncologic outcomes stratified by adherence to surveillance.

**Results:**

Eighty-five patients (54 male [63.5%]; median [IQR] age, 63.0 [54.0-73.0] years) were managed nonoperatively and followed for a median of 4.04 years (95% CI, 3.17-4.58 years). The 5-year overall survival was 82.3% (95% CI, 71.8%-94.5%), the 5-year disease-specific survival was 95.1% (95% CI, 89.6%-100.0%), the rate of local regrowth was 24.7% (21 patients), and the rate of distant metastases was 12.9% (11 patients). Among the 77 patients with more than 6 months of follow-up in their first year of surveillance, only 39.0% of patients (30 patients) achieved NCCN-recommended surveillance in the first year, and this decreased to 15.0% (3 patients) by year 5. However, the time to local regrowth and distant metastasis were similar regardless of the level of surveillance.

**Conclusions and Relevance:**

In this cohort study of patients with rectal cancer undergoing surveillance after nonoperative management, most did not achieve NCCN-recommended guidelines, although outcomes were not associated with surveillance intensity. This highlights the need for prospective evaluation of a surveillance regimen that is both feasible for patients and health care systems increasingly using nonoperative management. In addition, studies are warranted to explore patient preferences in rectal cancer care and to identify barriers to optimal surveillance.

## Key Points

**Question:**

What are the adherence rates to the National Comprehensive Cancer Network’s (NCCN) recommended surveillance guidelines for nonoperative management in patients with rectal cancer following a clinical complete response to neoadjuvant therapy?

**Findings:**

In this cohort study of 85 patients with rectal cancer who elected for nonoperative management between 2012 and 2023, only 39.0% of patients achieved all NCCN-recommended surveillance in the first year, and the percentage decreased to 15.0% by year 5.

**Meaning:**

These findings suggest that adherence to recommended surveillance guidelines was not achieved for most patients with rectal cancer undergoing nonoperative management protocols, highlighting the need for prospective evaluation of an optimal, yet feasible, surveillance regimen.

## Teaser

This cohort study examines adherence rates to National Comprehensive Cancer Network–recommended surveillance in patients undergoing nonoperative management for rectal cancer.

## Introduction

There is increasing utilization of nonoperative management (NOM) for locally advanced rectal cancer in the US.^1^ Patients prefer NOM,^2^ and numerous retrospective studies and clinical trials have shown that, although local regrowth rates can exceed 30%,^3,4,5^ identifying and addressing tumor regrowth promptly is safe with acceptable rates of salvage surgery and disease-specific survival outcomes.^4,5^ Despite guideline recommendations for intensive surveillance, there are minimal data assessing surveillance regimens in the 2 decades since NOM was first described.^6^

The 5-year surveillance protocols following oncologic resection for colorectal cancer have been studied extensively.^7,8^ However, for patients managed nonoperatively, surveillance regimens are guided by expert opinion and there are minimal data evaluating the safety and feasibility of surveillance programs. This gap partly arises from the origins of NOM as an opportunistic pathway for the minority of patients who achieved a clinical complete response (cCR) with long-course chemoradiation. Initially, the surveillance regimen included monthly digital rectal examinations, proctoscopy, biopsies, and serum carcinoembryonic antigen (CEA) monitoring, along with biannual abdominal or pelvic computed tomography (CT) scans and chest radiographs in the first year, with frequency adjustments thereafter.^6^ However, 15% of patients from that center were lost to follow-up, and there is no published information on adherence to the surveillance regimen.^3^ The existing recommended surveillance intervals emerged on the basis of patterns of tumor regrowth and modeling studies, suggesting, for instance, 3-month intervals for endoscopy and rectal magnetic resonance imaging (MRI) in the first year, followed by adjustments in subsequent years.^9^ This approach, adopted by the Organ Preservation in Patients with Rectal Adenocarcinoma (OPRA) trial, later informed the National Comprehensive Cancer Network (NCCN) guidelines.^4,10^ Despite the goal of early detection of local regrowth where salvage surgery is still possible, there is minimal evidence on the feasibility of surveillance in general population or clinical trial settings.

Currently, with use of multimodal neoadjuvant therapy, rates of cCR range from 40% to 80%, and ongoing trials are seeking to further increase this response rate, with the goal of increasing organ preservation.^11^ In this context, there is a pressing need for data regarding the feasibility of adhering to surveillance regimens for NOM of rectal cancer.^12^ Therefore, we evaluated NOM in one academic center, assessing both disease-related outcomes and adherence to recommended surveillance guidelines. We hypothesized that patients with rectal cancer who elect for NOM are unlikely to meet the recommended surveillance guidelines.

## Methods

This retrospective cohort study was approved by the University of Rochester Research Subjects Review Board and adheres to the Strengthening the Reporting of Observational Studies in Epidemiology (STROBE) reporting guidelines and standards for studies reporting clinical practice data.^13^ Two physicians (B.K.H.C. and A.L.) extracted additional information from the electronic medical records of patients identified through a prospectively maintained institutional database of patients with rectal cancer who elect for NOM. A waiver of informed consent was approved by the Research Subjects Review Board because the data were deidentified and the research posed minimal risk to participants, in accordance with 45 CFR §46.

### Study Population and Derivation of the Cohort

The study includes patients with clinical stage I to III rectal adenocarcinoma who underwent neoadjuvant therapy, had a cCR or near complete clinical response (nCR) at their first posttreatment endoscopic assessment, and opted for NOM between 2012 and 2023. All patients with an nCR at their posttreatment response assessment achieved a cCR within the first 3 months of surveillance. The decision to proceed with NOM was based on patient preference and multidisciplinary tumor board recommendations. We excluded patients who had transanal excisions before systemic therapy, those who declined oncologic resection, and patients with metastases. Our institution has been accredited through the National Accreditation Program for Rectal Cancer since 2017.

### Variables of Interest

We collected data regarding patient characteristics (age, sex, race, ethnicity, Eastern Cooperative Oncology Group Performance Status, insurance type, driving distance to cancer center, and Area Deprivation Index^14^), tumor (clinical TNM category, mismatch repair status, pretreatment CEA, and distance from the anal verge), treatment (clinical trial participation, treatment centers, type of neoadjuvant therapy, and posttreatment endoscopic response assessment), surveillance (dates of endoscopy, CEA, MRI, and CT of the chest, abdomen, or pelvis), salvage surgical procedures (pathologic stage [yp], surgery performed, and resection margin), and outcomes (local regrowth, distant metastases, second-line therapies [surgical resection, radiation, or chemotherapy], and vital status). Participants’ racial and ethnic identities were extracted from the electronic medical records and are included here for additional demographic context.

### Definitions

The neoadjuvant therapies used were total neoadjuvant therapy (≥6 cycles of induction or consolidation chemotherapy with FOLFOX [folinic acid, fluorouracil, and oxaliplatin], CAPEOX [oxaliplatin and capecitabine], or FOLFIRINOX [folinic acid, fluorouracil, irinotecan, and oxaliplatin] with long-course chemoradiotherapy [LCRT; 45-56 Gy over 25-28 fractions with concurrent capecitabine or fluorouracil]), short-course radiotherapy (SCRT; 25 Gy over 5 fractions) with chemotherapy, LCRT, and monotherapies (chemotherapy, dostarlimab, or SCRT alone).^15^

The posttreatment flexible sigmoidoscopy was the primary determinant of treatment response.^5,16^ A cCR was defined as no signs of ulceration, mass, or mucosal irregularities on the posttreatment restaging flexible sigmoidoscopy.^17,18^ An nCR was defined as stenosis, mucosal irregularities, small nodules, or superficial ulceration on the posttreatment restaging flexible sigmoidoscopy.^18,19^ Organ preservation was defined as the absence of low anterior resection or abdominoperineal resection with total mesorectal excision and no locoregional regrowth unless salvaged by R0 transanal excision.^19^

The NCCN surveillance guidelines and definitions used in this study are presented in Table 1. For each year of surveillance with at least 6 months of follow-up data starting from the date of the first posttreatment assessment, patients were classified as having ideal, adequate, or inadequate surveillance according to algorithms adapted from the postresection surveillance literature.^20,21^ In brief, achieving all NCCN criteria within a year was ideal surveillance. Adequate surveillance involved meeting the recommended number of endoscopies with at least 1 additional ideal level of radiographic or laboratory-based surveillance. Inadequate surveillance encompassed cases where there were insufficient endoscopies during the year or where ideal levels of endoscopy were achieved without other radiographic or laboratory surveillance components. In situations where patients were unable to undergo MRI surveillance owing to medical contraindications or intolerance, pelvic CT was considered as an alternative.^5^ Patients with less than 6 months of follow-up in a year were censored to reduce biases toward higher rates of inadequate surveillance as adherence was assessed in annual increments (eFigure 1 in Supplement 1).

**Table 1.** NCCN Surveillance Guidelines and Guideline-Concordant Surveillance Categorization

NCCN surveillance	For each year of surveillance with ≥6 mo of follow-up		
	Ideal surveillance	Adequate surveillance	Inadequate surveillance
Years 1-2: digital rectal examination and proctoscopy or flexible sigmoidoscopy every 3-4 mo; years 3-5: every 6 mo	Years 1-2: At least 3 endoscopies per year; years 3-5: at least 2 endoscopies per year (colonoscopy considered an endoscopy)	Ideal endoscopic surveillance and ≥1 other ideal levels of surveillance (MRI, CT, or CEA)	Insufficient endoscopic surveillance or ideal endoscopic surveillance with no other ideal levels of surveillance (MRI, CT, or CEA)
MRI of rectum every 6 mo for up to 3 y	Years 1-3: At least 2 rectal MRIs annually		
CT of chest and abdomen every 6-12 mo for 5 y; CT of pelvis once no longer doing MRI	Years 1-5: At least 1 CT of chest and abdomen annually, with pelvis after year 3		
CEA every 3-6 mo for 2 y, then every 6 mo for 5 y	Years 1-5: At least 2 CEAs annually		

Abbreviations: CEA, carcinoembryonic antigen; CT, computed tomography; MRI, magnetic resonance imaging; NCCN, National Comprehensive Cancer Network.

### Outcomes

The primary objective was to describe the clinical practice experience and adherence to guideline recommended NOM surveillance standards. Secondary objectives included assessing oncologic outcomes (local regrowth, distant metastases, overall survival, and disease-specific survival), identifying factors associated with undersurveillance, and comparing outcomes stratified by intensity of surveillance. When comparing outcomes of patients with ideal, adequate, and inadequate surveillance, patients were categorized on the basis of their adherence to surveillance in their first year.

### Statistical Analysis

Descriptive statistics are presented as the median and IQR for continuous variables and the frequency in percentage for categorical variables. The Mann-Whitney *U* and Kruskal-Wallis tests were used to compare continuous and ordinal variables, and categorical variables were compared using the χ^2^ test. Oncologic outcomes for the cohort were calculated as time from the date of completing treatment to the date of local regrowth, metastasis, or death. The 25th percentile and median survival time were estimated using the Kaplan-Meier method, and hypothesis testing was performed using the log-rank test with 95% CIs. In all primary surveillance adherence analyses, time zero was the date of the first posttreatment response assessment,^10^ as guidelines for timing the posttreatment endoscopic response assessment vary by the type of neoadjuvant treatment. Median follow-up was calculated from the treatment end date using the reversed Kaplan-Meier method.^22^

We performed several sensitivity analyses: first, we analyzed death as a competing risk for local regrowth or distant metastasis. Second, since patients with an nCR at their posttreatment assessment undergo more frequent endoscopic studies pending achievement of cCR, we excluded these patients from the evaluation of adherence. Third, given the widespread effects of the COVID-19 pandemic on care, we excluded surveillance for 1 year between March 2020 and March 2021. When 2 years of surveillance occurred within this period, the year with the majority between these dates was excluded. All statistics were performed using SAS statistical software version 9.4 (SAS Institute), RStudio software version 2023.03.0+ (R Project for Statistical Computing), and Prism software version 10 (GraphPad), with statistical significance considered a 2-tailed *P* < .05. Analysis was completed from March through May 2024.

## Results

Eighty-five patients with clinical stage I to III rectal cancer (54 male [63.5%]; median [IQR] age, 63.0 [54.0-73.0] years) were followed for a median of 4.04 years (95% CI, 3.17-4.58 years), for 304 total person-years (Table 2). Most were White (82 patients [96.5%]), 3 patients (3.5%) were Black, and 3 patients (3.5%) were of Hispanic ethnicity. Overall, 26 patients (30.6%) participated in a clinical trial and 24 (28.2%) had fragmented care, receiving portions of their care (radiation or medical oncology, or colorectal surgery) at separate facilities. Most received total neoadjuvant therapy (52 patients [61.2%]), SCRT with chemotherapy (18 patients [21.2%]), and LCRT (12 patients [14.1%]). Posttreatment endoscopic response assessments occurred a median (IQR) of 6.9 (5.0-10.0) weeks after completing neoadjuvant therapy, and 61 patients (71.8%) had a cCR at the posttreatment response assessment whereas 24 patients (28.2%) initially had an nCR before achieving a cCR at a subsequent assessment.

**Table 2.** Features of Cohort and Comparison by Adherence to Surveillance

Characteristic	Patients, No. (%)^a^				*P* value
	Total cohort (N = 85)	Surveillance^b^			
		Inadequate (n = 29)	Adequate (n = 18)	Ideal (n = 30)	
Age at diagnosis, median (IQR), y	63.0 (54.0-73.0)	63.0 (58.0-73.0)	64.5 (60.3-73.5)	57.5 (50.0-67.5)	.05
Sex					
Female	31 (36.5)	14 (48.3)	2 (11.1)	12 (40.0)	.03
Male	54 (63.5)	15 (51.7)	16 (88.9)	18 (60.0)	
Race					
Black	3 (3.5)	1 (3.4)	0	2 (6.7)	.51
White	82 (96.5)	28 (96.5)	18 (100.0)	28 (93.3)	
Hispanic ethnicity	3 (3.5)	0	0	0	NA
Distance to cancer center from residence, median (IQR), miles	23.7 (11.3-65.8)	25.2 (11.5-51.0)	20.2 (10.5-39.6)	36.0 (14.1-89.1)	.16
National ADI, median (IQR)	71.5 (55.1-83.7)	71.8 (62.1-85.1)	69.2 (54.7-78.8)	72.5 (55.1-83.4)	.38
State ADI, median (IQR)	8.9 (8.0-9.6)	8.9 (8.1-9.7)	8.8 (7.9-9.3)	9.0 (7.8-9.6)	.54
Care at separate facilities	24 (28.2)	11 (37.9)	7 (38.9)	5 (16.7)	.13
Insurance					
Government (Medicare or Tricare)	34 (40.0)	14 (48.3)	10 (55.6)	8 (26.7)	.11
Medicaid or uninsured	11 (12.9)	6 (20.7)	1 (5.6)	4 (13.3)	
Private	40 (47.1)	9 (31.0)	7 (38.9)	18 (60.0)	
Eastern Cooperative Oncology Group Performance Status					
0	59 (69.4)	19 (65.5)	12 (66.7)	24 (80.0)	.41
1	26 (30.6)	10 (34.5)	6 (33.3)	6 (20.0)	
Clinical trial participant	26 (30.6)	6 (20.7)	5 (27.8)	13 (43.3)	.16
Mismatch repair deficient	3 (3.5)	2 (6.9)	0	0	.33
Clinical T category					
cT1	2 (2.4)	1 (3.4)	0	0	.26
cT2	15 (17.6)	4 (13.8)	1 (5.6)	8 (26.7)	
cT3	54 (63.5)	19 (65.5)	15 (83.3)	17 (56.7)	
cT4	12 (14.1)	5 (17.2)	1 (5.6)	4 (13.3)	
Missing	2 (2.4)	0	1 (5.6)	1 (3.3)	
Clinical N category					
N0	19 (22.4)	4 (13.8)	2 (11.1)	7 (23.3)	.33
N1	47 (55.3)	15 (51.7)	12 (66.7)	19 (63.3)	
N2	19 (22.4)	10 (34.5)	4 (22.2)	4 (13.3)	
Distance from anal verge, median (IQR), cm	6.0 (4.3-10.0)	5.5 (3.1-7.6)	7.3 (4.1-10.9)	5.3 (4.5-9.8)	.71
Tumor distance from anal verge, cm					
<5	36 (42.4)	12 (41.4)	6 (33.3)	14 (46.7)	.16
5-10	36 (42.4)	15 (51.7)	6 (33.3)	12 (40.0)	
>10	13 (15.3)	2 (6.9)	6 (33.3)	4 (13.3)	
Type of neoadjuvant therapy					
Total neoadjuvant therapy	52 (61.2)	18 (62.1)	9 (50.0)	20 (66.7)	.02
Long-course chemoradiation	12 (14.1)	2 (6.9)	7 (38.9)	2 (6.7)	
Short-course radiation plus chemotherapy	18 (21.2)	8 (27.6)	1 (5.6)	8 (26.7)	
Short-course radiation alone	1 (1.2)	1 (3.4)	0	0	
Chemotherapy alone	1 (1.2)	0	1 (5.6)	0	
Immunotherapy alone	1 (1.2)	0	0	0	
Time from completing therapy to endoscopic response assessment, median (IQR), wk	6.9 (5.0-10.0)	8.0 (5.7-12.3)	8.0 (5.1-9.9)	5.9 (3.9-8.1)	.37
Initial endoscopic response to therapy					
Complete clinical response	61 (71.8)	24 (82.8)	9 (50.0)	22 (73.3)	.05
Near complete clinical response	24 (28.2)	5 (17.2)	9 (50.0)	8 (26.7)	
Recurrence					
Local regrowth	21 (24.7)	5 (17.2)	4 (22.2)	6 (20.0)	.91
Distant metastasis	11 (12.9)	5 (17.2)	4 (22.2)	1 (3.3)	.12
Time to local regrowth, median (IQR), y	0.9 (0.4-1.6)	0.8 (0.6-0.9)	1.3 (0.9-1.8)	1.7 (1.2-2.1)	.11
Time to distant metastasis, median (IQR), y	1.9 (1.6-2.3)	1.9 (1.8-2.2)	1.9 (1.7-2.6)	3.2 (3.2-3.2)	.47
Total follow-up, median (95% CI), y	4.04 (3.17-4.58)	3.53 (2.53-4.08)	4.87 (2.81)^c^	4.67 (3.17-5.54)	.04
Vital status at the last follow-up					
Dead	11 (12.9)	5 (17.2)	5 (27.8)	1 (3.3)	.11
Alive	70 (82.4)	23 (79.3)	12 (66.7)	29 (96.7)	
Unknown or lost to follow-up	4 (4.7)	1 (3.4)	1 (5.6)	0	

Abbreviations: ADI, Area Deprivation Index; NA, not applicable.

^a^ Percentages may not add up to 100% owing to rounding.

^b^ Patients included in the comparison of intensity of surveillance analysis had a minimum of 6 months of surveillance follow-up. Therefore, 7 patients from the 85 were excluded.

^c^ The upper boundary of the 95% CI is missing because of a never observed failure time for which the upper boundary of the 95% CI is less than 5%.

### Oncologic Outcomes

**Figure 1.**
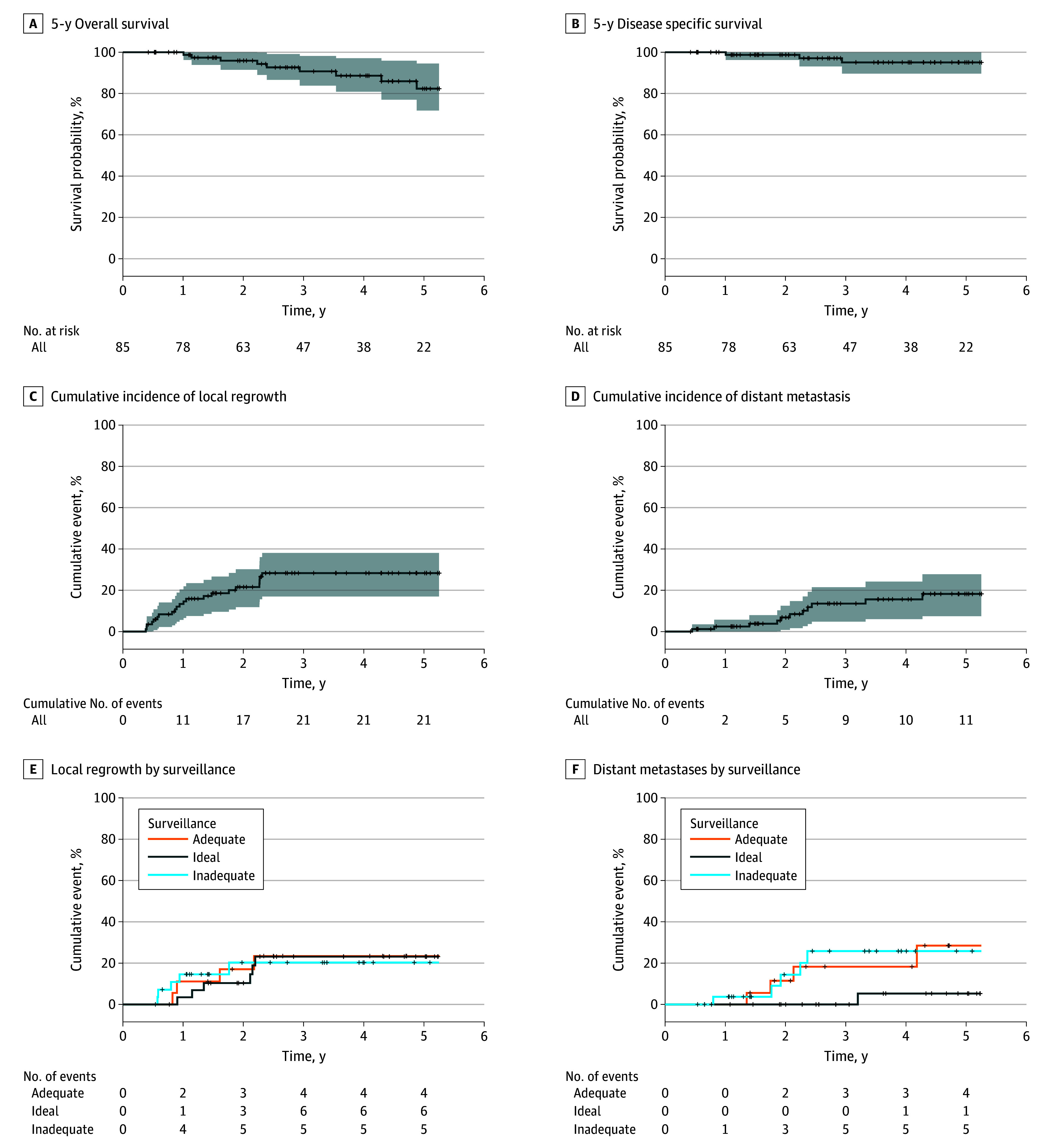
Oncologic Outcomes Graphs show oncologic outcomes for the cohort at 5 years. Overall survival was 82.3% (A), disease-specific survival was 95.1% (B), and the estimated rate of local regrowth at 3 years was 28.3% (C), with an estimated rate of distant metastases at 5 years of 18.2% (D). Time to local regrowth (E) and distant metastasis (F) are shown by adherence to surveillance (ideal, adequate, and inadequate).

The 5-year overall survival was 82.3% (95% CI, 71.8%-94.5%), and the 5-year disease-specific survival 95.1% (95% CI, 89.6%-100.0%) (Figures 1A and 1B). Local regrowth occurred in 21 of 85 patients (24.7%), with a 3-year estimated rate of 28.3% (95% CI, 17.0%-38.1%) (Figure 1C) and median (IQR) time to local regrowth of 10.8 (6.3-19.8) months. Distant metastases occurred in 11 of 85 patients (12.9%), with a 5-year estimated rate of 18.2% (95% CI, 7.4%-27.8%) (Figure 1D). Of the patients with distant metastases, 7 (33.3%) had metastases after local regrowth, whereas 4 patients (6.3%) had metastases without local disease.

### Adherence to Surveillance

**Figure 2.**
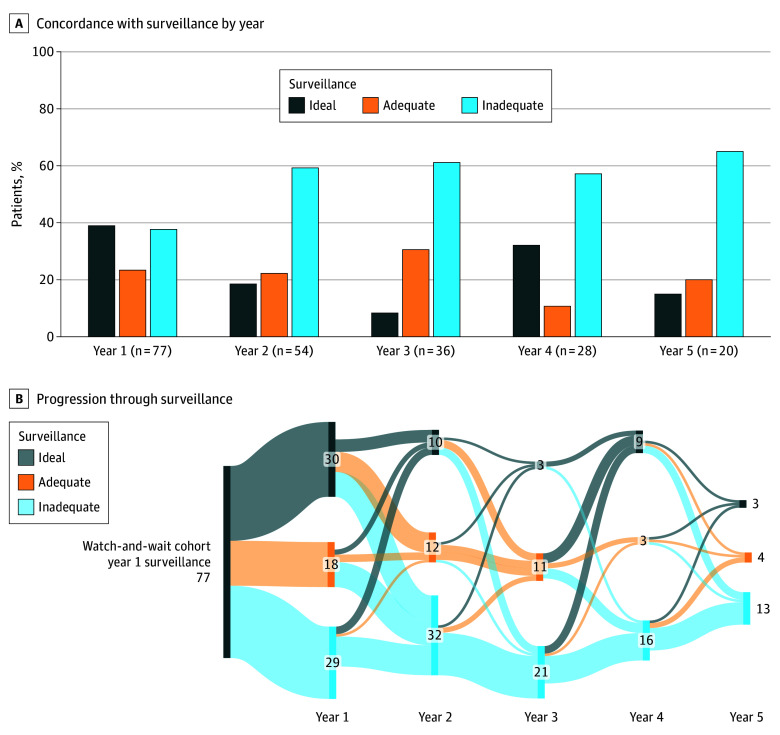
Concordance with Surveillance Guidelines Graphs show overall adherence to National Comprehensive Cancer Network–recommended screening guidelines in 77 eligible patients. Overall, ideal adherence to guidelines declined by year (A). Patients did not necessarily fall into a single category for adherence by year, and instead often transitioned between ideal and inadequate levels of surveillance (B).

Seventy-seven patients, each with more than 6 months of follow-up in their first year of surveillance, were eligible for the analysis of adherence to surveillance guidelines (Table 2). Patients who met criteria for ideal surveillance were younger, whereas patients who had adequate surveillance were more often male and more likely to have received LCRT (Table 2). In the first year, 39.0% of patients (30 patients) had ideal surveillance, which decreased to 18.5% (10 patients) in the second year and to 15.0% (3 patients) by year 5 (Figure 2A). Adequate surveillance was observed in 23.4% of patients (18 patients) in the first year, and 22.2% (12 patients) in the second year (Figure 2A). Throughout the surveillance period, no patients maintained ideal surveillance, 4 maintained at least adequate surveillance, and 4 maintained inadequate surveillance (Figure 2B).

**Figure 3.**
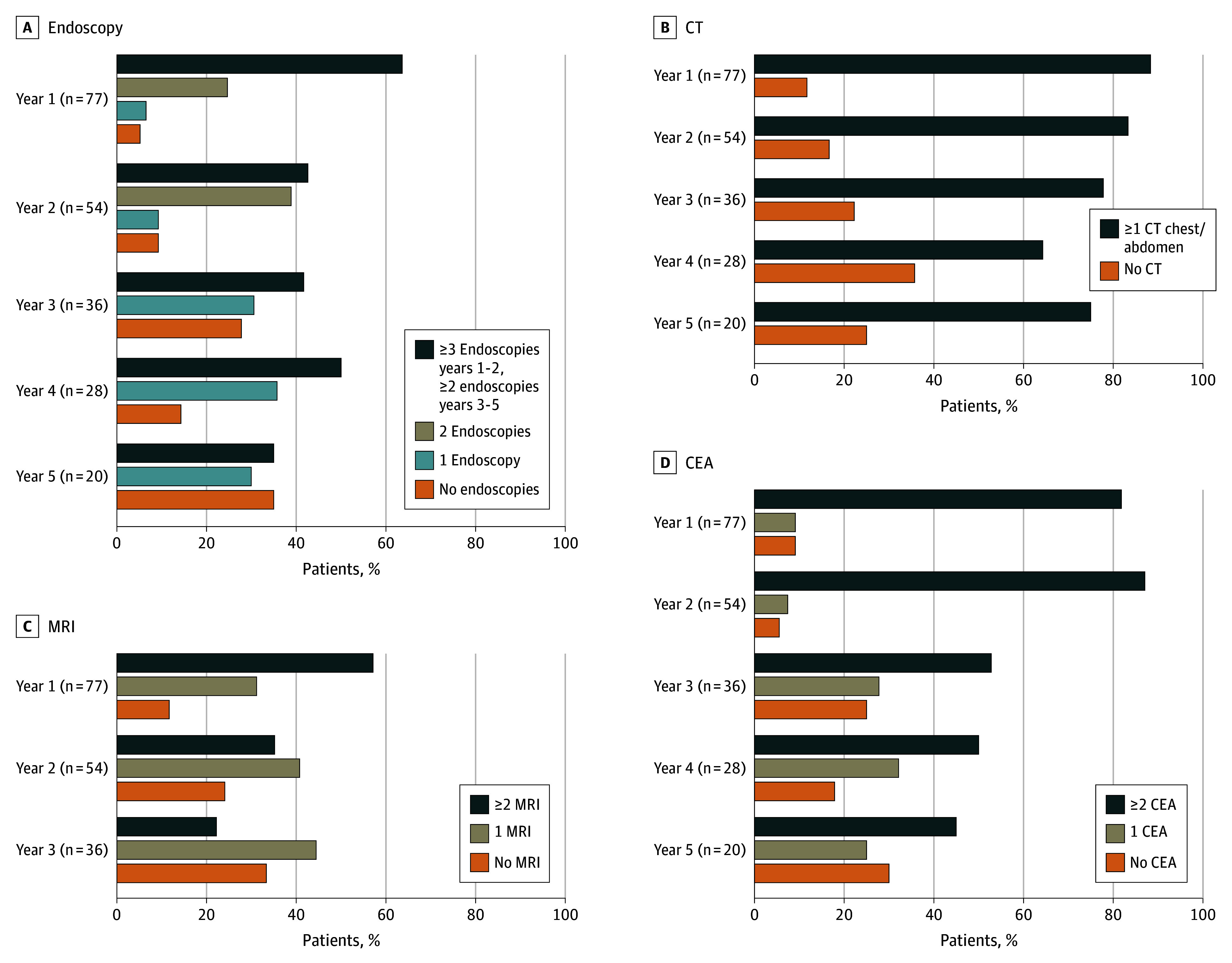
Percentage of Patients Who Satisfied National Comprehensive Cancer Network Guidelines by Imaging Modality Graphs show percentages of patients with ideal, adequate, and inadequate surveillance by year and by surveillance modality, including endoscopy (A), computed tomography (CT; B), magnetic resonance imaging (MRI; C), and carcinoembryonic antigen (CEA; D).

The adherence to each surveillance modality varied by the test and the year of follow-up (Figure 3). In the first year, 57.1% of patients (44 patients) achieved the recommended 2 annual MRIs, which decreased to 35.2% (19 patients) the second year. Although 63.6% of patients (49 patients) underwent a minimum of 3 endoscopies in the first year, this percentage decreased to 42.6% (23 patients) in the second year; however, 88.3% (68 patients) had at least 2 endoscopies in the initial year (Figure 3). Notably, 88.3% of patients (68 patients) obtained at least 1 CT chest and abdomen scan in the first year, maintaining the highest adherence percentage throughout the study period (Figure 3).

### Surveillance and Outcomes

The time to local regrowth (median [IQR], 1.7 [1.2-2.1] years for ideal surveillance, 1.3 [0.9-1.8] years for adequate surveillance, and 0.8 [0.6-0.9] years for inadequate surveillance) and distant metastasis (median [IQR], 3.2 [3.2-3.2] years for ideal surveillance, 1.9 [1.7-2.6] years for adequate surveillance, and 1.9 [1.8-2.2] years for inadequate surveillance) was similar despite intensity of surveillance (Figures 1E and 1F). In addition, the ability to achieve an R0 resection, rates of ostomy formation, and subsequent survival outcomes did not differ by the intensity of surveillance (eTable in Supplement 1).

### Sensitivity Analyses

When patients who initially had an nCR at posttreatment assessment were excluded, given the likelihood of more frequent endoscopies until achieving a cCR, the distribution of patients achieving ideal (40.0% [22 patients]), adequate (16.4% [9 patients]), and inadequate surveillance (43.6% [24 patients]) in the first year was similar (eFigure 2A in Supplement 1). In addition, excluding the period of the COVID-19 pandemic, the percentage of patients meeting ideal (43.3% [26 patients]), adequate (25.0% [15 patients]), and inadequate surveillance (31.7% [19 patients]) in the first year of surveillance was similar (eFigure 2B in Supplement 1).

## Discussion

The oncologic outcomes of patients in this institutional cohort study align with those reported in the literature, while also revealing 3 key findings. First, achieving full adherence to the NCCN guidelines is challenging in a clinical practice setting. Second, endoscopies had one of the lowest rates of adherence when analyzing each surveillance modality independently, although 88.3% of patients managed to have them twice per year. Third, there was no difference in the time to detect local tumor regrowth or distant metastases between patients according to their intensity of surveillance. In addition, regardless of intensity of surveillance, patients who required salvage resections had similar resection margins, ostomy formation rates, and subsequent survival outcomes despite intensity of surveillance. To our knowledge, this study represents the largest cohort of patients managed nonoperatively evaluating adherence to NCCN surveillance recommendations—a crucial aspect of this treatment strategy given the significantly higher rate of local regrowth compared with patients who undergo upfront surgery.

Advocates of NOM emphasize the importance of surveillance, yet no centers have reported adherence rates to surveillance in the context of historical cohorts or clinical trials. Currently, no prospective studies have evaluated the optimal surveillance regimen for patients on NOM protocols, nor are there data on the ability to achieve, or the consequences of not following, recommended surveillance. In our cohort, fewer than 40% of patients met the NCCN-recommended surveillance levels, with none achieving ideal surveillance over all 5 years. Only 4 patients maintained at least adequate surveillance throughout follow-up, indicating that most transition between adequate and inadequate surveillance annually. Furthermore, our study highlights the challenges of obtaining timely endoscopies and MRIs. Despite suboptimal adherence to NCCN guidelines, the 5-year oncologic outcomes in our cohort align with the literature.^3,5^ The time to local recurrence and the salvage surgical outcomes were similar across surveillance levels. Although this finding must be interpreted with caution because of the small sample size, it may suggest an opportunity to revise surveillance protocols without sacrificing long-term outcomes. Modeling studies^9^ that emphasize intensive surveillance during the highest risk periods for local regrowth, combined with our data showing which surveillance modalities have the highest adherence rates, can guide the development of clinical trials testing alternate surveillance regimens for patients who achieve cCR. This approach could quickly identify evidence-based surveillance regimens that are more feasible for patients without compromising long-term outcomes.

The current surveillance guidelines for NOM have parallels in other high-risk populations requiring intensive endoscopic surveillance. Patients with familial adenomatous polyposis require frequent endoscopic surveillance for the detection of high-risk gastrointestinal lesions, and one study^23^ found that up to 1 in 4 high-risk patients were underadherent to screening guidelines. Elsewhere, a meta-analysis^24^ found that adherence to North American guideline recommendations was only 54.6% after high-risk polypectomies, with increased rates of late surveillance colonoscopies, leading to potential increased risk of postcolonoscopy colorectal cancer. The challenge of adherence to screening recommendations is widespread and well-described, yet patients undergoing NOM for rectal cancer are advised to follow time-intensive and resource-intensive surveillance regimens.

### Limitations

The findings of this study should be considered with certain limitations. There is no reference standard for surveillance, and our institution participated in the OPRA trial, which began in 2013 and informed the current NCCN guidelines. In addition, the current NCCN guidelines were published in 2023, although they were used in our study because they are the most contemporarily relevant in the US. Moreover, while these guidelines are recent, they were heavily influenced by the OPRA clinical trial protocol screening guidelines,^4^ which have been publicly available since 2012. Although this is a relatively large cohort of patients with rectal cancer managed initially nonoperatively, some analyses are limited by a small event size. Next, this study partly overlaps with the COVID-19 pandemic, which disrupted patient care globally. To account for this, we excluded any surveillance during a 1-year period from March 2020 to March 2021, at which point our institution had reinstated access to all surveillance modalities, and our results were similar.

## Conclusions

In this cohort study of patients with rectal cancer who elected NOM at an academic center, we highlight a failure to meet current screening recommendations. As NOM continues to be increasingly utilized, centers must track surveillance, monitor oncologic outcomes, and develop a shared data repository allowing for the ongoing evaluation of the safety and outcomes as these patients are increasingly cared for outside of the clinical trial setting in many disparate centers. Finally, these findings highlight a need for high-quality qualitative studies to address patient priorities in rectal cancer care and barriers to surveillance. In a watch-and-wait scenario, we must actively watch or surveil patients and not risk just waiting in order to offer patients organ preservation after rectal cancer therapy with acceptable oncologic outcomes.

## Notes

Supplement 1. **eTable**. Clinical Characteristics and Outcomes of Patients With Local Regrowth **eFigure 1.** Consort Diagram for Derivation of Cohort **eFigure 2.** Results of Sensitivity Analyses

Supplement 2.
Data Sharing Statement
