# Supplementary material for: Challenges of Surveillance in Implementing Nonoperative Management for Rectal Cancer
Source: JAMA Netw Open. 2024 Dec 3;7(12):e2448682. doi: 10.1001/jamanetworkopen.2024.48682 (PMC11615709; doi:10.1001/jamanetworkopen.2024.48682)
Supplement: Supplement 1. — eTable. Clinical Characteristics and Outcomes of Patients With Local Regrowth eFigure 1. Consort Diagram for Derivation of Cohort eFigure 2. Results of Sensitivity Analyses [file jamanetwopen-e2448682-s001.pdf]

Supplemental Online Content

Hilty Chu BK, Loria A, Dhimal T, et al. Challenges of surveillance in implementing nonoperative management for rectal cancer. *JAMA Netw Open*. 2024;7(12):e2448682. doi:10.1001/jamanetworkopen.2024.48682

eTable. Clinical Characteristics and Outcomes of Patients With Local Regrowth

eFigure 1. Consort Diagram for Derivation of Cohort

eFigure 2. Results of Sensitivity Analyses

This supplemental material has been provided by the authors to give readers additional information about their work.

© 2024 Hilty Chu BK et al. *JAMA Network Open.*

eTable. Clinical Characteristics and Outcomes of Patients With Local Regrowth

Patient	Surveillance	Salvage Operation	Height above Anal Verge (cm)	Initial Clinical Stage	Pathologic Stage	Resection Margin	Pelvic / Stomal Recurrence	Distant Metastases	Disease Status
1	Ideal	LAR	>10	cT3N1	ypT3N0	R0	No	Yes, liver	DOC
2	Ideal	None	>10	cT3N1	NA	NA	NA	Yes, liver	DOC
3	Ideal	APR	<5	cT3N0	ypT2N1	R0	Yes, re-excised	No	NED
4	Ideal	APR (aborted)	>10	cT3N1	NA	NA	NA	No	DOD
5	Ideal	APR	>10	cT3N2	ypT2N0	R0	No	No	NED
6	Ideal	LE[a]	>10	cT3N2	ypT2N0	R0	No	No	AWD[a]
7	Ideal	APR	<5	cT2N1	ypT2N0	R0	No	Yes, lung	AWD
8	Adequate	APR	<5	cT3N1	ypT3N1	R0	No	No	NED
9	Adequate	LAR	5-10	cT3N1	ypT2N0	R0	No	No	NED
10	Adequate	APR	<5	cTXN1	ypT2N1	R0	No	No	NED
11	Inadequate	LAR	5-10	cT4N1	ypT2N2	R0	No	No	UNK
12	Inadequate	APR	<5	cT3N1	ypT4N0	R0	No	Yes, liver	DOD
13	Inadequate	LAR	5-10	cT3N2	ypT1N0	R0	No	No	NED
14	Inadequate	LAR	5-10	cT4N1	ypT4N0	R0	Yes, re-excised	Yes	AWD
15	Inadequate	None	<5	cT3N1	NA	NA	NA	Yes, lung/liver	DOD
16	Excluded	APR	5-10	cT3N0	ypT2N0	R0	No	No	UNK
17	Excluded	LAR	>10	cT4N1	ypT3N0	R0	No	No	NED
18	Excluded	LAR	5-10	cT3N0	ypT3N0	R0	No	No	NED
19	Excluded	LAR	>10	cT2N0	ypT2N0	R0	No	Yes, lung[b]	NED
20	Excluded	APR	<5	cT1N0	ypT3N0	R0	No	No	NED
21	Excluded	Pending[c]	>10	cT3N2	Pending	Pending	No	No	AWD[c]

Abbreviations: LAR, low anterior resection; APR, abdominoperineal resection; LE, local excision; DOC, died of other causes; NED, no evidence of disease; DOD, died of disease; AWD, alive with disease; UNK, unknown (lost to follow-up).

[a] Patient awaiting surgical date for TME
[b] Patient underwent lung wedge resection, now NED.
[c] Patient awaiting surgical date for TME

© 2024 Hilty Chu BK et al. *JAMA Network Open.*

eFigure 1: Consort Diagram for Derivation of Cohort

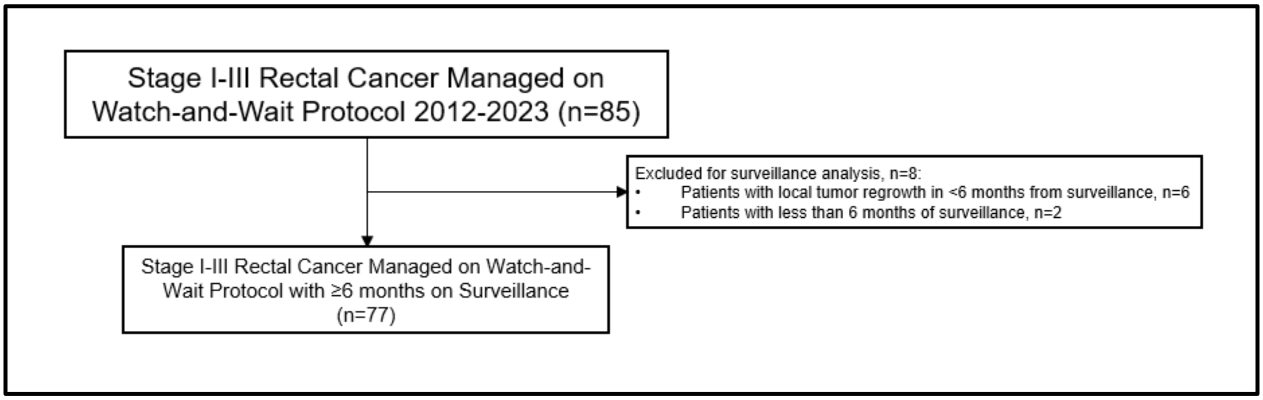

Figure legend: Derivation of cohort for analysis of adherence to surveillance.

© 2024 Hilty Chu BK et al. *JAMA Network Open.*

eFigure 2: Results of Sensitivity Analyses

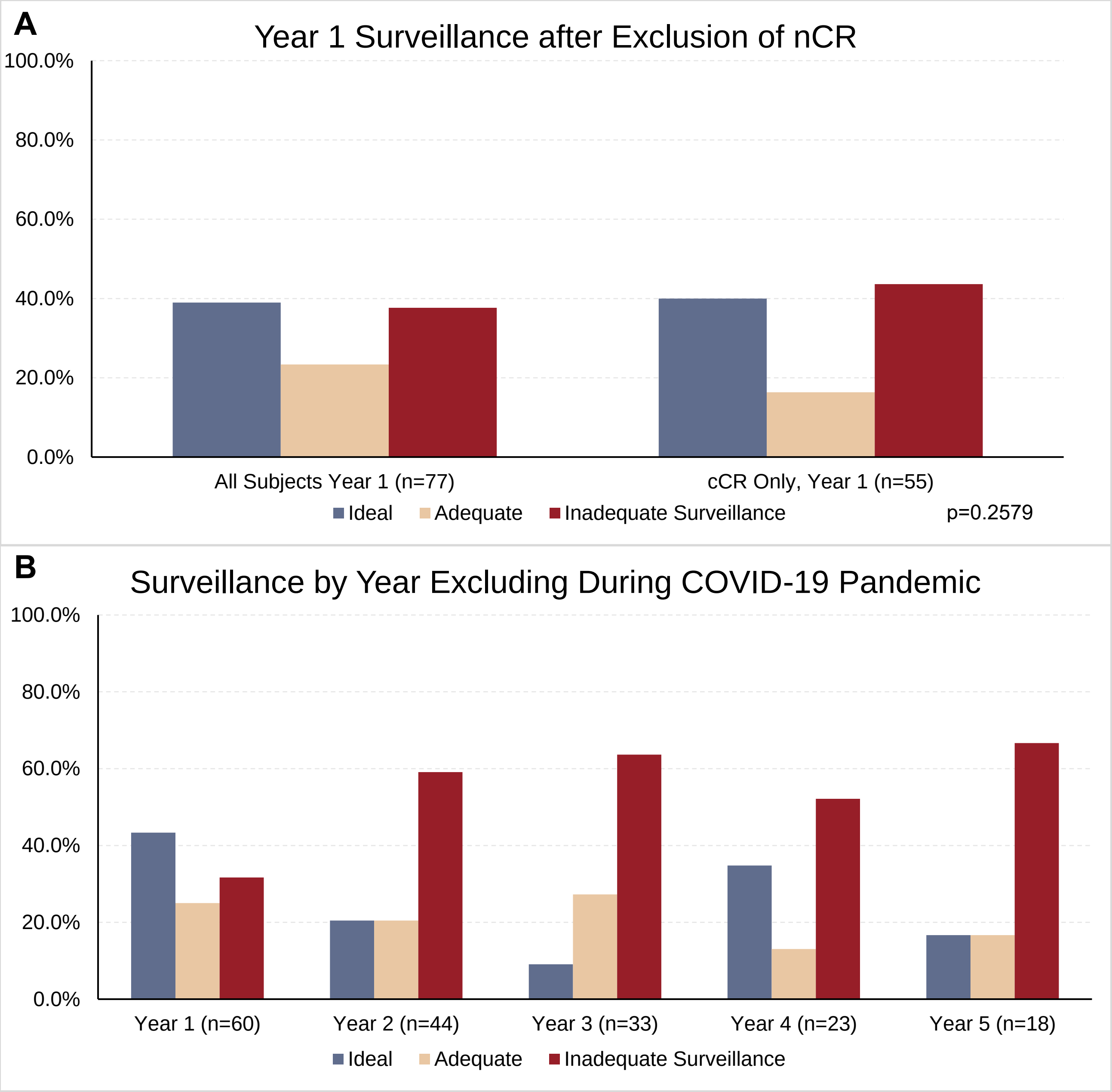

Figure legend: Results of the sensitivity analyses. After excluding patients with nCR at initial endoscopic assessment, the percent of patients categorized as 'ideal', 'adequate', and 'inadequate' was not significantly changed (Panel A). After excluding surveillance during the COVID-19 Pandemic, the distribution of patients into each surveillance category remained similar (Panel B).

© 2024 Hilty Chu BK et al. *JAMA Network Open.*
