# Supplementary material for: Challenges of Surveillance in Implementing Nonoperative Management for Rectal Cancer
Source: JAMA Netw Open. 2024 Dec 3;7(12):e2448682. doi: 10.1001/jamanetworkopen.2024.48682 (PMC11615709; doi:10.1001/jamanetworkopen.2024.48682)
Supplement: Supplement 2. — Data Sharing Statement [file jamanetwopen-e2448682-s002.pdf]

Data Sharing Statement

Hilty Chu. Challenges of Surveillance in Implementing Nonoperative Management for Rectal Cancer. *JAMA Netw Open*. Published December 03, 2024.
doi:10.1001/jamanetworkopen.2024.48682

Data
Data available: Yes
Data types: Deidentified participant data
How to access data: Proposals and required review documentation should be directed to Ila_Marianetti@urmc.rochester.edu.
When available: With publication

Supporting Documents
Document types: None

Additional Information
Who can access the data: Deidentified data will be made available to investigators who provide a methodologically sound proposal.
Types of analyses: Data will be made available for collaborative analyses.
Mechanisms of data availability: Deidentified data will be made available to investigators who provide a methodologically sound proposal which has been approved by an independent review committee. To gain access, researchers will also need to sign a data access agreement.
